# Supplementary material for: A novel method for the injection and manipulation of magnetic charge states in nanostructures
Source: Sci Rep. 2016 Sep 12;6:32864. doi: 10.1038/srep32864 (PMC5018726; doi:10.1038/srep32864)
Supplement: Supplementary Information [file srep32864-s1.pdf]

Supplementary Information

Title: **A Novel Method for the Injection and Manipulation of Magnetic Charge States in Nanostructures**

Authors: J. C. Gartside, D. M. Burn, W. R. Branford and L. F. Cohen

Brief description of the supplementary simulation videos showing the time evolution of various dynamic micromagnetic processes. All videos were produced using the object-oriented micromagnetic framework (OOMMF).

A) Injection of CCW chirality 360° DW by an upwards-moving magnetic charge
B) Injection of CW chirality 360° DW by a downwards-moving magnetic charge

These videos correspond to the injection process described in figure 2.

C) Tip-mediated collapse of existing 360° DW followed by re-injection

This video corresponds to the collapse and re-injection process described in figure 4.

D) Spatial manipulation of existing 360° DW

This video illustrates the movement of an existing 360° DW by a weaker magnetic charge. Corresponding data is shown in figure 6.
